# Electricity-Based Locoregional Cancer Therapies and Their Associated Drug Delivery Technologies: From Principles of Action to Clinical Implications

**DOI:** 10.34133/bmr.0386

**Published:** 2026-07-07

**Authors:** Ngoc-Thuan Truong, Arjaree Jobdeedamrong, Hye-Jin Yoo, Ratchapol Jenjob, Seokyoung Bang, Su-Geun Yang

**Affiliations:** ^1^ Department of Biomedical Science, BK21 FOUR Program in Biomedical Science and Engineering, Inha University College of Medicine, Incheon 22212, Republic of Korea.; ^2^ Department of Biomedical Engineering, Dongguk University, Goyang 10326, Republic of Korea.

## Abstract

Electric field-based therapies are rapidly emerging as innovative modalities in the fight against cancer, offering targeted, minimally invasive, and often immunomodulatory alternatives to conventional treatments. This review comprehensively examines the current landscape and prospects of electricity-based locoregional cancer therapies and their associated drug delivery technologies. Key modalities, including irreversible electroporation, tumor-treating fields, radiofrequency ablation, electrochemotherapy, and iontophoresis, are discussed regarding their mechanisms of action, clinical applications, and integration with immunotherapy. In addition, emerging strategies such as smart electro-responsive drug carriers and integrative therapeutic systems are highlighted as promising approaches for achieving electrically controlled, site-specific, and precision-guided drug delivery. Although electricity-based locoregional cancer therapies show promise, challenges such as variable tissue responses, device optimization, and long-term safety remain. Current research aims to address these issues and enhance outcomes through combinations with immunotherapy. By drawing on advances in bioelectricity, nanotechnology, and immunology, these therapies have the potential to substantially improve the precision and personalization of cancer treatment.

## Introduction

Cancer continues to pose a major global health burden, with conventional treatments such as surgery, chemotherapy, and radiotherapy often limited by nonspecific toxicity, poor tumor penetration, and the development of resistance [1]. These limitations have driven the development of innovative therapeutic strategies aimed at improving specificity, efficacy, and personalization in cancer care.

While surgical resection remains the gold standard for many solid tumors, its applicability is often limited by factors such as tumor location, size, and proximity to critical anatomical structures [2,3]. Tumors situated in complex regions—particularly those abutting major blood vessels, bile ducts, nerves, or deep-seated organs like the pancreas and liver—may be considered unresectable or carry a high surgical risk [4,5]. In this context, electricity-based locoregional therapies offer important advantages. These minimally invasive approaches enable precise tumor targeting while preserving surrounding critical structures and reducing procedural morbidity [6], making them particularly suitable for tumors in anatomically challenging locations where conventional surgical or thermal approaches are not feasible [7,8].

Electricity-based locoregional cancer therapies have emerged as a promising class of modalities that utilize controlled electric fields to target tumors with high precision [9]. Techniques such as irreversible electroporation (IRE), tumor-treating fields (TTFields), and radiofrequency ablation (RFA) can disrupt tumor cell membranes, induce immunogenic cell death (ICD), and modulate the tumor microenvironment, offering both direct cytotoxic effects and the potential to stimulate antitumor immunity [1,9]. Electrochemotherapy (ECT) further combines electric pulses with chemotherapeutic agents to enhance intracellular drug uptake and therapeutic efficacy [10–12].

A noteworthy advancement in these fields is iontophoresis. Iontophoresis is a technique that utilizes low-intensity electric currents to drive charged or polar drugs across biological membranes, such as the skin, for enhanced drug delivery [13]. This technique enables localized, noninvasive, and controlled drug delivery, overcoming barriers to drug penetration in solid tumors and reducing systemic toxicity. Iontophoresis is gaining attention for its ability to achieve higher local drug concentrations at tumor sites, complementing other electricity-based interventions [14–16].

Emerging strategies such as smart electro-responsive drug carriers have attracted increasing attention as complementary platforms for achieving electrically controlled and site-specific drug delivery. These carriers are engineered to release therapeutic agents in response to externally applied electrical stimuli, enabling on-demand, site-specific, and controlled drug delivery [17]. Such systems can be tailored to respond to specific electrical parameters, providing a level of control and personalization unattainable with conventional drug delivery platforms [1]. The integration of these smart carriers with wearable or implantable electro-stimulating devices further enhances the potential for real-time and patient-specific cancer therapy [18].

Beyond direct tumor ablation and improved drug delivery, electricity-based locoregional therapies—including iontophoresis and smart electro-responsive carriers—are increasingly recognized for their ability to modulate the immune system and enhance the efficacy of immunotherapies. By promoting ICD and facilitating the release of tumor antigens, these approaches may help convert immunologically “cold” tumors into “hot” ones, thereby improving responses to immune checkpoint inhibitors and other immunomodulatory agents [19].

Despite these advances, challenges remain in optimizing treatment parameters, standardizing devices, and ensuring long-term safety [19]. Ongoing research is focused on addressing these issues and exploring synergistic combinations with other therapeutic modalities.

To provide a clearer conceptual framework for these diverse approaches, it is important to distinguish the functional roles of electrical stimulation based on the magnitude and biological effects of the applied electric field. High-intensity electric fields, as used in modalities such as IRE and RFA, primarily induce direct tumor cell death through irreversible membrane disruption or thermal damage [20–22]. In contrast, intermediate electric fields are employed in approaches such as ECT and TTFields, where electrical stimulation induces biological effects without immediate tissue ablation—ECT enhances intracellular drug uptake via transient membrane permeabilization [23,24], while TTFields disrupt mitotic processes and inhibit tumor cell proliferation [24–26]. Low-intensity electrical stimulation, as used in iontophoresis and smart electro-responsive drug carriers, enables controlled drug transport or electrically triggered release without causing marked tissue injury [27,28]. In addition, electricity-based cancer therapies can also be understood across multiple technological layers, including device placement (e.g., invasive electrodes or external field-generating arrays), electric field generation and control, and the resulting biological or therapeutic effects [29,30]. While these layers are interrelated, distinguishing them conceptually helps clarify the diverse mechanisms and applications of these technologies. Together, these perspectives highlight that electricity-based approaches represent fundamentally distinct yet complementary therapeutic strategies.

This review provides a comprehensive overview of electricity-based locoregional cancer therapies, with particular emphasis on tumor ablation therapy and iontophoresis while also discussing emerging strategies such as smart electro-responsive drug carriers and integrative therapeutic systems. We discuss their mechanisms of action, technological innovations, clinical applications, and future directions, highlighting their transformative potential in advancing precision oncology and personalized cancer treatment.

## Electricity-Based Locoregional Cancer Therapy

Electricity-based locoregional cancer therapies have emerged as innovative treatment modalities offering localized and minimally invasive approaches to tumor eradication. IRE uses high-voltage pulses to destroy cancer cells while sparing nearby structures [1,31]. TTFields disrupt tumor cell division with low-intensity electric fields, protecting healthy tissue [1,25]. In contrast, RFA destroys tumors by generating localized heat [21,32,33]. Collectively, these locoregional cancer therapies selectively target cancer cells, minimize systemic toxicity, and may boost immune responses to enhance immunotherapy [22,31,34–39]. Their growing clinical use highlights their promise for treating both superficial and deep-seated solid tumors with precision and safety.

### Irreversible electroporation

**Fig. 1.**
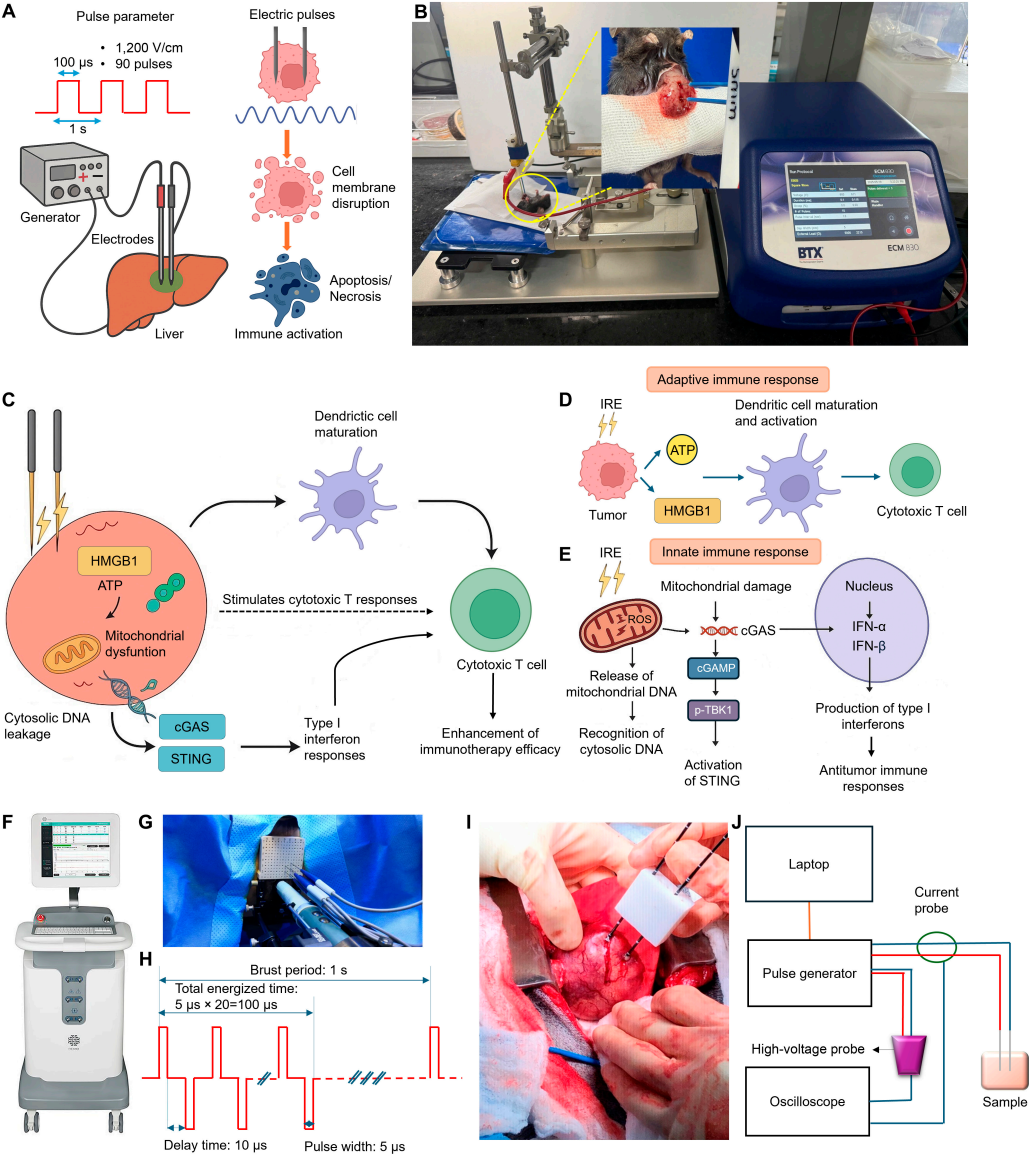
Schematics for IRE/H-FIRE treatment setup and immunogenic effects of IRE in cancer therapy. (A) Illustration of the IRE procedure: Electrical pulses generated by a pulse generator are delivered through electrodes inserted into a liver tumor, leading to disruption of tumor cell membranes and subsequent cell death via apoptosis or necrosis, as well as immune activation. (B) Photographs of the IRE system. The placement of electrodes in the liver cancer of a mouse (inset). (C) IRE induces immunogenic cell death (ICD) through mitochondrial dysfunction and cytosolic DNA leakage, leading to the release of DAMPs, then activates the cGAS–STING pathway, promotes type I interferon responses, and stimulates dendritic cell maturation, enhancing cytotoxic T cell activation and boosting immunotherapy efficacy. (D) Adaptive immune: IRE also engages the adaptive immune response by facilitating dendritic cell activation and subsequent stimulation of cytotoxic T cells, contributing to systemic antitumor immunity. (E) Innate immune: IRE induces mitochondrial damage and reactive oxygen species (ROS) generation, leading to the release of mitochondrial DNA. Cytosolic DNA activates the cGAS-STING pathway, resulting in type I interferon production and antigen-presenting cell activation, driving antitumor immune responses. Panels (A) and (C) to (E) were generated using ChatGPT (GPT-4o-mini) and further revised by the authors. The use of this AI tool is fully disclosed in accordance with journal guidelines. All rights to the generated content are retained by the authors, and the tool’s terms of use permit commercial use. (F) The composite steep pulse therapeutic apparatus (REMEDINE, Shanghai, China). (G) Electrode needles are placed through a brachytherapy template grid and the perineum. Reproduced with permission from Ref. [221]. (H) The schematic of high-frequency bipolar pulses (created using Microsoft PowerPoint, Office 2016). (I) Picture depicting the insertion of the bipolar electrodes into a canine lung tumor. (J) Schematic diagram of the H-FIRE experimental circuit setup. Reproduced with permission from Ref. [222].

IRE is an emerging nonthermal ablation technique used in oncology to treat solid tumors, especially those located near vital structures such as blood vessels, bile ducts, and nerves (Fig. 1A and B). Unlike conventional thermal ablation, IRE uses pulsed electric fields to induce cell death while preserving the extracellular matrix and critical anatomical features [40–42].

#### Mechanism of action

IRE works by delivering high-voltage, short-duration electric pulses (typically 1,000 to 3,000 V/cm; 10 to 100 μs) through needle electrodes strategically placed in or around the tumor (Fig. 1B, inset) [43]. These pulses create permanent nanopores in the cell membrane, disrupting membrane integrity and cellular homeostasis. This leads to regulated forms of cell death, including apoptosis and necrosis, without causing severe thermal injury to surrounding tissues (Fig. 1A) [44–46]. The preservation of connective tissue and vascular structures is a key advantage, enabling safe treatment of tumors in challenging anatomical locations [47,48].

#### Immunogenic effects

Recent research has highlighted the ability of IRE to induce ICD (Fig. 1C) [49]. This process involves the release of damage-associated molecular patterns (DAMPs) such as high mobility group box 1 (HMGB1) and adenosine triphosphate (ATP), which stimulate dendritic cell maturation and cytotoxic T-cell responses, potentially leading to systemic antitumor immunity (Fig. 1D) [34,36,37,50–54]. Additionally, IRE can cause mitochondrial dysfunction and cytosolic DNA leakage, activating innate immune pathways like cGAS (cyclic GMP-AMP synthase)–STING (stimulator of interferon genes) and promoting type I interferon responses, mechanisms that may enhance the efficacy of immunotherapies (Fig. 1E) [55–58].

#### Advances in electroporation techniques

In addition to standard IRE, sublethal and high-frequency electroporation techniques are being explored. Sublethal IRE can facilitate drug delivery or activate cellular stress pathways [59–61], while high-frequency irreversible electroporation (H-FIRE) uses short bipolar pulses at megahertz frequencies to minimize muscle contractions [62–64] and improve selectivity for malignant cells [65,66]. H-FIRE has shown efficacy in preclinical models of brain and liver tumors (Fig. 1F to J) [67–69].

#### Clinical applications

Table S1 shows the current clinical application and trials for IRE. IRE has demonstrated particular value in the management of locally advanced pancreatic cancer (LAPC), where surgical resection is often not possible. Clinical studies have shown that IRE, especially when combined with chemotherapy, can achieve a median overall survival exceeding 24 months in LAPC patients [70]. Despite these promising results, recurrence at the ablation margins remains a challenge [71], prompting ongoing research into combination therapies with immune checkpoint inhibitors and chemotherapeutics [40,72,73].

#### Current clinical issues

Despite its advantages, IRE faces several important challenges and limitations in clinical practice. One major issue is the heterogeneity of tissue response to electric fields, which can result in incomplete ablation and tumor recurrence, particularly at the margins of the treated area [74,75]. Variability in tissue conductivity and the intrinsic voltage tolerance of different tumor cells make it difficult to ensure uniform cell death throughout the target region [76]. Additionally, real-time assessment of treatment efficacy remains problematic; current imaging modalities such as computed tomography (CT) and magnetic resonance imaging are not always capable of providing immediate feedback on the completeness of ablation, increasing the risk of under- or overtreatment [77].

Another concern is the relatively high retreatment rate, with studies reporting rates ranging from 8% to 36.6% for prostate cancer [78], and the occurrence of out-of-field recurrences due to the multifocal nature of some tumors and limitations in preprocedural imaging or biopsy strategies [79]. While IRE is generally associated with low rates of major complications, adverse events such as urinary tract infections and hematuria and, more rarely, severe events like arterial hemorrhage or cardiac arrest have been reported, particularly in complex cases or when treating tumors adjacent to major vessels [80].

Functionally, while IRE preserves urinary continence and erectile function in most prostate cancer patients, some decline in sexual function has been observed, and longer-term oncological outcomes remain under investigation [78,81]. Lastly, the short follow-up times and heterogeneity in study designs and patient selection criteria limit the ability to draw definitive conclusions about long-term safety and efficacy. Ongoing research is focused on optimizing electric field delivery, improving real-time monitoring, and developing combination strategies with immunotherapies or oncolytic viruses to overcome these limitations and enhance the therapeutic potential of IRE [76,77].

To overcome these limitations and further enhance the therapeutic efficacy and immunomodulatory potential of IRE, recent studies have increasingly explored its integration with advanced therapeutic platforms. Notably, Han et al. developed CpG-ODN-functionalized metal–phenolic network immunoactive nanoparticles and combined them with bipolar IRE, demonstrating a synergistic strategy for cancer immunotherapy. In this system, the nanoparticles promote dendritic cell activation and M1 macrophage polarization, while IRE induces tumor antigen release through ICD, together leading to enhanced CD8^+^ cytotoxic T-cell-mediated antitumor immunity [82]. In addition, a recent study by our group demonstrated that nanoparticle-assisted IRE using gold-doped mesoporous silica nanoparticles can appreciably enhance membrane permeabilization, increase reactive oxygen species generation, and improve tumor ablation efficacy [83]. Beyond nanoparticle-based strategies, Park et al. [84] demonstrated that combining IRE with advanced immunotherapies, such as chimeric antigen receptor natural killer (CAR-NK) cell therapy, can further enhance antitumor immune responses and improve tumor control in hepatocellular carcinoma models. Importantly, emerging clinical evidence indicates that combining IRE with immunotherapy leads to improved therapeutic outcomes compared to IRE alone in patients with LAPC, highlighting the translational potential of these combination strategies. Furthermore, multifunctional platforms incorporating imaging capabilities offer opportunities for image-guided IRE and real-time monitoring of treatment responses, enabling improved spatial precision and treatment control [85,86]. Collectively, these advances highlight the emerging potential of integrating electroporation-based tumor ablation with nanotechnology and immunotherapy to achieve synergistic, immune-activating, and precision-guided cancer therapy.

### Tumor-treating fields

TTFields therapy is a locoregional, noninvasive anticancer treatment that uses a portable device to deliver alternating electric fields to tumors via transducer arrays placed on the patient’s skin over the tumor site. This allows for targeted therapy while sparing surrounding healthy tissues and critical anatomical structures [87,88].

#### Mechanism of action and immunogenic effects

**Fig. 2.**
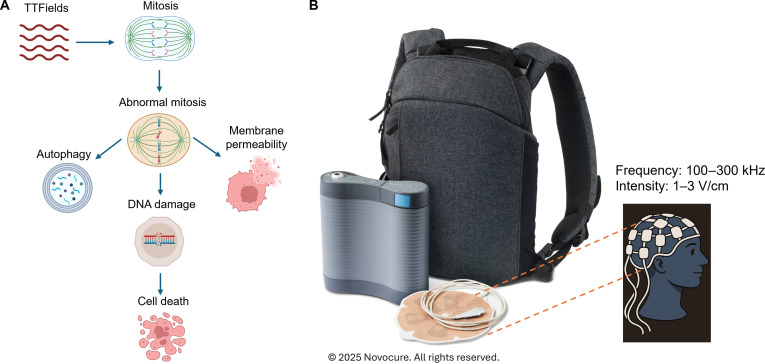
Principles of action for TTFields and clinical products. (A) Schematic illustration of TTFields mechanism: TTFields disrupt cancer cell division by interfering with mitotic spindle formation during anaphase, leading to abnormal mitosis. This results in increased autophagy, membrane permeability, and DNA damage, ultimately causing cell death. This figure was created with BioRender.com. The use of BioRender is fully acknowledged in accordance with the journal’s guidelines. All rights to the figure content are retained by the authors. (B) The Optune Gio product: a wearable, portable, and FDA-approved medical device used to treat glioblastoma multiforme. Reproduced with permission from © 2025 Novocure GmbH—all rights reserved (https://www.novocure.com). The model part was generated using Perplexity AI and revised by authors (version as of June 2025). The use of this AI tool is fully disclosed in accordance with journal guidelines. All rights to the generated content are retained by the authors, and the tool’s terms of use permit commercial use.

TTFields are low-intensity (1 to 3 V/cm), intermediate-frequency (100 to 300 kHz) alternating electric fields to disrupt cancer cell division (Fig. 2B) [25,38,89]. They interfere with mitosis by disrupting the mitotic spindle and causing abnormal chromosome segregation, ultimately leading to cell cycle arrest and apoptosis [38,90,91]. Additional effects include autophagy induction, impaired DNA repair, increased membrane permeability, inhibition of cell migration, and disruption of the blood–brain barrier, all of which contribute to their anticancer efficacy (Fig. 2A) [87,92,93].

In addition to their direct antimitotic effects, TTFields have been shown to stimulate antitumor immunity. Preclinical and clinical data indicate that TTFields can induce ICD, leading to increased infiltration of cytotoxic T cells and the activation of adaptive immune responses [94]. When combined with immune checkpoint inhibitors, such as anti-PD-1 or anti-CTLA-4 antibodies [95], TTFields have demonstrated synergistic effects, resulting in greater tumor volume reduction and enhanced immune activation in vivo [38,96]. These findings support the potential of TTFields as a platform for combination immunotherapy, particularly in tumors with otherwise limited responsiveness to immune-based treatments [35].

#### Advances in TTFields techniques

Research has expanded the application of TTFields beyond glioblastoma multiforme (GBM), with studies exploring optimal field frequencies, array placements, and combination strategies [25]. High-frequency TTFields and integration with modern systemic therapies are being tested to maximize efficacy and minimize toxicity. Additionally, TTFields have been shown to increase cell membrane permeability and may enhance the delivery and effectiveness of chemotherapeutic agents [93].

#### Clinical applications and development

TTFields have received regulatory approval for the treatment of GBM and malignant pleural mesothelioma and are under investigation for a range of other solid tumors, including non-small cell lung cancer [97] and pancreatic cancer [98]. In a recent Phase 3 trial (PANOVA-3), TTFields in combination with chemotherapy improved survival in patients with locally advanced, unresectable pancreatic adenocarcinoma, marking the first positive study for this challenging patient population [87]. Ongoing trials are also evaluating TTFields in breast, endometrial, and renal cell carcinomas, as well as in combination regimens with chemotherapy and immunotherapy [25]. An overview of TTFields in clinical trials is summarized in Table S2.

#### Current clinical issues

Despite these advances, TTFields therapy faces several challenges. Patient adherence is an important issue, as optimal efficacy requires wearing the device for at least 18 hours per day, yet real-world data indicate that many patients fall short of this target [99]. Skin irritation at array sites is common and can affect compliance [87]. Additionally, anatomical variability and differences in tumor conductivity can lead to heterogeneous field distribution and variable treatment responses [87]. Long-term data beyond 3 years are limited, and the best strategies for integrating TTFields with other systemic therapies remain under investigation. Ongoing and future trials aim to address these limitations and clarify the role of TTFields in broader cancer care [38,87].

To overcome these limitations and further enhance the therapeutic potential of TTFields, increasing attention has been directed toward its integration with advanced drug delivery systems, nanotechnology, and immunotherapy. Nanoparticle-based platforms have been utilized to improve the delivery of chemotherapeutic agents and sensitizers under TTFields exposure, resulting in increased tumor cell susceptibility and improved treatment outcomes [100]. Furthermore, TTFields have been investigated in combination with immunotherapy, where they may enhance antitumor immune responses by promoting cellular stress and immunogenic signaling [101,102]. These combinatorial approaches highlight the potential of TTFields as part of multimodal treatment strategies that integrate physical and biochemical mechanisms for improved cancer therapy [103].

### Radiofrequency therapy in cancer

Radiofrequency (RF) therapy is a well-established modality in cancer treatment that delivers electromagnetic energy within the frequency range of 100 kHz to 1 MHz to biological tissues. The application of RF energy induces oscillating electric fields, resulting in ionic agitation and resistive heating. These thermal effects primarily lead to coagulative necrosis when high-power levels are employed [104]. Clinically, RF therapy is most frequently associated with RFA, a standard technique for treating solid tumors such as those found in the liver, lung, kidney, bone, and pancreas [21]. However, growing interest has emerged around low-intensity, nonablative RF approaches that aim to modulate tumor biology without causing thermal injury [105].

#### Thermal RFA

**Fig. 3.**
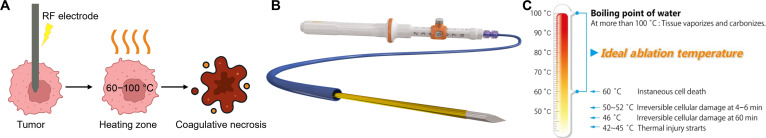
Mechanism of action for RFA and RFA device for clinical applications. (A) Schematic illustration of the mechanism of RFA. RF energy is delivered through an electrode, heating the target tissue to 60 to 100 °C and inducing coagulative necrosis within the ablation zone. This figure was created with BioRender.com. The use of BioRender is fully acknowledged in accordance with the journal’s guidelines. All rights to the figure content are retained by the authors. (B) Image of a commercial endobiliary RFA catheter (EUSRA TaeWoong Medical, USA) used for minimally invasive thermal ablation. (C) Thermal ablation temperature scale: cell death occurs rapidly at ≥60 °C, with varying degrees of irreversible damage and thermal injury depending on exposure temperature and duration. Panels (B) and (C) were reproduced with permission from TaeWoong Medical USA and STARmed official (https://taewoongusa.com).

RFA involves the image-guided insertion of a needle electrode into the tumor, typically under CT or ultrasound assistance, followed by the application of high-frequency alternating current. This current heats the surrounding tissue to temperatures between 60 and 100 °C, resulting in direct thermal destruction of tumor cells via coagulative necrosis (Fig. 3A to C) [106]. RFA is particularly beneficial for patients with inoperable tumors or those who are poor surgical candidates due to comorbidities or tumor location. Clinical outcomes have shown RFA to be highly effective in achieving local tumor control with minimal invasiveness. For instance, in patients with hepatocellular carcinoma, RFA is recognized as a first-line treatment for tumors smaller than 3 cm, providing outcomes comparable to surgical resection [107].

Despite its efficacy, RFA has several limitations. These include the risk of incomplete ablation at tumor margins, the heat-sink effect where adjacent blood flow dissipates thermal energy and limited engagement of systemic antitumor immunity [108]. In response to these challenges, current research is exploring combination therapies in which RFA is paired with immune checkpoint inhibitors, chemotherapy, or electroporation [21,32]. These combinatorial strategies aim to trigger more robust local and systemic anticancer responses, enhancing the overall therapeutic outcome [39].

#### Nonthermal and low-intensity RF for cancer modulation

In recent years, the scope of RF therapy has expanded beyond thermal ablation. Emerging research has focused on nonthermal RF applications, which operate at lower power and aim to influence tumor signaling pathways without inducing cell death through heat. One such modality is capacitive resistive electric transfer (CRET), which applies RF currents (0.45 to 0.6 MHz) to alter cancer cell behavior, including proliferation, apoptosis, and immune signaling, without notable tissue heating [109,110].

#### Immune modulation effects of thermal and nonthermal RF therapies

Both thermal and nonthermal RF therapies exert immune-modulatory effects, albeit via distinct mechanisms. Thermal ablation via RFA leads to ICD by releasing DAMPs [111–113]. These molecules activate antigen-presenting cells and stimulate antitumor T-cell responses [22,114]. Although the immune activation triggered by RFA can generate systemic effects, it is often insufficient to control metastatic disease without adjunctive treatments [115,116].

#### Recent developments and clinical trials

Several advancements in RF therapy have expanded its potential in cancer treatment, moving beyond local tumor ablation to systemic therapeutic strategies [21] (Table S3). One important development is the integration of RFA with immunotherapy [33]. Clinical studies are currently investigating the safety and efficacy of combining RFA with anti-PD-1 immune checkpoint inhibitors in liver cancer patients [117,118]. This approach aims to harness the immunogenic effects of thermal ablation while simultaneously enhancing immune activation against residual or metastatic disease. Additionally, wearable RF technologies are being explored to enable chronic, noninvasive treatment [119,120].

To further improve therapeutic outcomes and overcome current limitations of RFA, recent studies have explored its integration with advanced nanotechnology, biomaterials, and immunotherapy strategies. For instance, nanoparticle-assisted RFA systems have been developed to improve heat localization and tumor destruction, while simultaneously enabling drug delivery or immune activation. In particular, combinations of RFA with immunotherapeutic strategies, such as immune checkpoint inhibitors, have demonstrated enhanced systemic antitumor responses through the promotion of ICD and antigen release [121,122]. Additionally, smart biomaterials, particularly injectable and thermosensitive hydrogels have been employed to achieve localized and sustained drug release following RFA, further improving treatment efficacy and reducing tumor recurrence [123]**.**

## Electricity-Based Locoregional Drug Delivery

Although electricity-based locoregional cancer therapies such as IRE, TTFields, and RFA have achieved notable clinical success, their broader efficacy remains constrained by several limitations, including incomplete tumor eradication due to heterogeneous electric field distribution and challenges in treating irregular tumor margins [92,124,125]. In addition, their ability to deliver therapeutic agents uniformly into dense tumor tissues or across biological barriers remains limited [126,127]. To overcome these challenges, increasing attention has been directed toward complementary electricity-based drug delivery approaches that enhance the transport or controlled release of therapeutic agents through the application of electrical stimuli. These include ECT, which enhances intracellular drug uptake via transient membrane permeabilization [23,128], and iontophoresis, which drives charged molecules across biological barriers [14,129].

These methods offer precise, site-specific drug delivery with reduced systemic exposure, especially valuable in targeting difficult-to-access tumors or crossing tight biological barriers like the blood–brain barrier [130,131].

### ECT (combined therapy of electricity and chemotherapy)

**Fig. 4.**
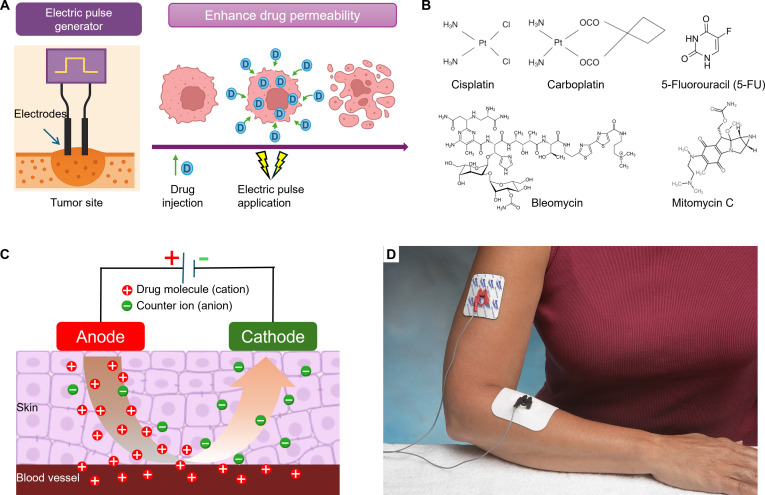
Mechanism and commonly used chemotherapeutic agents in ECT. (A) Schematic of the ECT mechanism: following intratumoral drug administration, electric pulses are applied to the tumor site using needle electrodes, transiently increasing cell membrane permeability and enhancing drug uptake, ultimately leading to cancer cell death. (B) Chemical structures of commonly used chemotherapeutic agents in ECT, including platinum-based drugs (cisplatin and carboplatin), antimetabolites (5-fluorouracil), glycopeptides (bleomycin), and alkylating agents (mitomycin C). Schematic and clinical application of iontophoresis for transdermal drug delivery. (C) Schematic diagram of iontophoresis-based transdermal drug delivery. An iontophoresis device generates a low electrical current that drives charged drug ions through the skin and into the underlying blood vessels via active transport, enhancing drug absorption. Panels (A) to (C) were created with BioRender.com and ChatGPT (GPT-4o-mini) and further revised by the authors. The use of BioRender is fully acknowledged in accordance with the journal’s guidelines. The use of this AI tool is fully disclosed in accordance with journal guidelines. All rights to the generated content are retained by the authors, and the tool’s terms of use permit commercial use. (D) Clinical setup of an iontophoresis device applied to a patient’s forearm, showing electrode placement and connection to the iontophoresis controller. Reproduced with permission from North Coast Medical, Inc. (https://www.ncmedical.com/).

ECT is a minimally invasive cancer treatment that combines the administration of chemotherapeutic agents with the application of pulsed electric fields to enhance drug uptake and tumor cell death (Fig. 4A) [132,133]. ECT is primarily used for cutaneous [133,134] and subcutaneous tumors [135], such as basal cell carcinoma (BCC), squamous cell carcinoma (SCC), and melanoma, and head and neck skin cancer [136,137], but its application is expanding to deep-seated malignancies [12,138,139]. The technique is particularly advantageous for patients who are elderly [140], have comorbidities, or are resistant to conventional therapies, as it offers effective tumor control with minimal systemic toxicity and can be performed in outpatient settings [141–143].

#### Enhanced local accumulation of chemotherapeutic agents

ECT operates by delivering high-voltage electric pulses (typically 100 to 1,000 V/cm, 50 to 100 μs) [144] to tumor tissue following the administration of hydrophilic chemotherapeutic agents such as bleomycin or cisplatin (Fig. 4B) [142,145]. These electric pulses induce reversible electroporation, transiently increasing cell membrane permeability and allowing the chemotherapeutic drugs to enter tumor cells at concentrations far higher than would be possible by passive diffusion alone. This results in enhanced cytotoxicity, substantial tumor necrosis, and apoptosis. Additionally, ECT disrupts tumor vasculature, causing a “vascular lock” [146,147] effect that prolongs drug retention within the tumor and further increases therapeutic efficacy [148,149].

#### Immunogenic effects

Beyond direct cytotoxicity, ECT has been shown to promote immunologically recognizable forms of cell death, including necrosis and ICD [150]. Preclinical and clinical studies demonstrate that ECT with agents like bleomycin, oxaliplatin, or cisplatin leads to substantial tumor necrosis and the release of immunogenic markers, which activate dendritic cells and cytotoxic T lymphocytes [142]. This immune activation may contribute to the observed abscopal effects, where localized ECT induces systemic antitumor responses, and may explain improved outcomes in immunocompetent patients compared to immunodeficient ones [10]. Such immune stimulation has prompted research into combining ECT with immune checkpoint inhibitors and gene electrotransfer for further therapeutic benefit [11,142,146].

#### Advances in ECT techniques

Recent advances in ECT include the development of high-frequency, less painful electroporation protocols, the use of novel electrode designs for hard-to-reach tumors [151], and the integration of gold nanoparticles to further enhance drug delivery and reduce side effects [152]. Real-time imaging and electric impedance tomography are being explored to optimize electric field distribution and treatment precision [153,154]. Calcium electroporation, which uses calcium ions instead of chemotherapeutic drugs, is also under investigation as a nontoxic alternative with promising results [146,155,156].

#### Clinical applications

Table S4 summarizes current clinical trials for ECT. ECT is highly effective for superficial tumors, with objective response rates ranging from 75% to 99% in BCC, SCC, and melanoma [31,138]. For example, a recent study in elderly patients with BCC reported comparable recurrence rates between standard and reduced-dose bleomycin, suggesting that ECT is a viable option for those with limited life expectancy [143]. Another clinical trial found a 100% overall response rate (51% complete, 49% partial) in skin cancer patients 1 month after ECT, with BCC showing the highest complete response rate [138]. ECT is generally well-tolerated, with pain being the most common side effect, and it can be repeated or combined with other modalities as needed. Ongoing research is extending ECT to deep-seated tumors, including pancreatic cancer, where it has demonstrated improved local control and quality of life in select patients [157,158].

#### Current clinical issues

Despite its benefits, ECT faces several challenges. Protocol standardization remains a concern, as treatment parameters and drug dosages can vary between centers [128,159]. There is also a need for multidisciplinary teams and specialized equipment, which limits widespread adoption. While most adverse effects are mild and transient, such as pain and local inflammation, recurrence can occur, particularly with reduced drug dosages or in immunocompromised patients [143]. Quality-of-life studies indicate that ECT is generally well-tolerated and can improve symptoms such as pain and bleeding, but long-term data and larger multicenter trials are needed to further define its role in oncology [143,151,158,160].

To address these limitations and further enhance the clinical potential of ECT, recent studies have explored its integration with advanced drug delivery systems including immunotherapeutic strategies and nanocarrier-based formulations that have been developed to improve the stability and tumor targeting of chemotherapeutic agents used in ECT, thereby increasing intracellular drug accumulation following electroporation [11,152,161]. In addition, ECT has been shown to synergize with immunotherapy by inducing ICD and promoting the release of tumor-associated antigens, which can enhance systemic immune responses when combined with immune checkpoint blockade [162]. These findings support the role of ECT as a versatile platform for combination therapies in both local and systemic cancer treatment.

### Iontophoresis

Iontophoresis is a noninvasive technique that uses a low-intensity electric current to enhance the penetration of charged therapeutic agents through biological membranes (Fig. 4C and D). Iontophoresis has emerged as a promising strategy for targeted cancer treatment [13,163].

#### Mechanism of iontophoresis

Iontophoresis enhances drug delivery through 2 primary mechanisms: electro-repulsion and electroosmosis. Electro-repulsion involves the direct movement of charged drug molecules under the influence of an electric field, whereas electroosmosis refers to the convective flow of solvent, carrying neutral or weakly charged molecules along with it [164]. Additionally, the application of an electric current can transiently increase the permeability of biological membranes, facilitating deeper drug penetration into tissues [14].

#### Clinical trials

In the context of cancer therapy, iontophoresis has been employed to deliver chemotherapeutic agents—such as cisplatin, 5-fluorouracil (5-FU), methotrexate, and doxorubicin (DOX)—directly to tumor sites. This localized drug delivery strategy is designed to enhance intratumoral drug concentration while minimizing systemic distribution, thereby improving therapeutic efficacy and reducing adverse effects [164–167]. Preclinical studies have provided compelling evidence for this approach (Table S5). For instance, 5-FU delivered via iontophoresis demonstrated elevated local concentrations and improved therapeutic outcomes in SCC xenografts [165].

Moreover, researchers have explored the integration of iontophoresis with nanoparticle-based carriers to improve drug loading, targeting, and controlled release [166,168–170]. Liposomal formulations of DOX delivered through iontophoresis have exhibited superior tumor penetration and greater antitumor efficacy compared to conventional passive diffusion methods [171]. Clinical applications are also emerging. A particularly innovative advancement includes the development of an implantable iontophoretic device (ACT-IOP-003) by Focal Medical, designed to administer gemcitabine directly to pancreatic tumors [172]. This technology aims to maximize local drug exposure while limiting systemic toxicity.

#### Current issues and challenges

Despite the encouraging progress in preclinical and early clinical studies, several challenges limit the widespread application of iontophoresis in cancer therapy. One of the primary limitations is the restricted penetration depth, as iontophoresis is most effective for delivering drugs to superficial or easily accessible tumors, such as those in the skin, oral cavity, or breast [16,129,173,174]. Deeper-seated or internal tumors, like those in the pancreas or liver, require invasive adaptations or implantable systems, which remain under development [16,129]. Additionally, device optimization is crucial; ensuring consistent current delivery, safety, and patient comfort in both wearable and implantable systems remains a technical hurdle [175]. Poorly regulated or prolonged current application can cause skin irritation, burns, or tissue damage, especially at higher current densities [176]. Another major barrier is the lack of standardized protocols—there is no consensus on current intensity, treatment duration, or drug formulations suitable for iontophoretic cancer treatment, which hampers reproducibility across studies and complicates regulatory approval [14,177]. Furthermore, the complex regulatory pathway for device–drug combination products poses an additional obstacle, requiring simultaneous evaluation of both safety and efficacy under stringent frameworks. Finally, challenges related to drug solubility, tissue retention, and off-target effects persist, especially in the heterogeneous tumor microenvironment, which can affect electrokinetic transport and drug response. Addressing these challenges will require interdisciplinary innovation in bioengineering, materials science, and oncology to ensure safe, scalable, and clinically effective iontophoretic systems for cancer therapy [174].

## Future Technology and Prospects

Despite the clinical progress of electricity-based locoregional therapies, including IRE, TTFields, and RFA, several limitations remain, including heterogeneous electric field distribution leading to incomplete tumor ablation, insufficient penetration of therapeutic agents into dense tumor tissues, and limited control over drug release and localization [99,178,179]. Addressing these limitations requires not only refinement of existing technologies but also the development of next-generation strategies. Compared to established modalities (Table S6), emerging strategies such as electro-responsive drug carriers aim to address these limitations. In this context, emerging approaches such as smart electro-responsive drug carriers, along with advances in materials science, device engineering, and integrative therapeutic systems, are expected to play a critical role in shaping the future of precision and personalized cancer therapy [180–182].

### Smart electro-responsive drug carriers

**Fig. 5.**
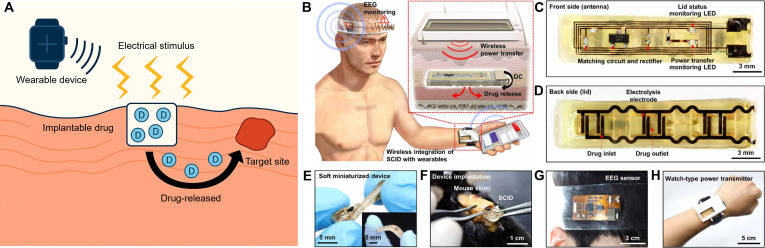
Electro-responsive smart drug delivery systems and wireless integration for cancer therapy. (A) Conceptual illustration of a smart electro-responsive drug delivery system. Electrically responsive nanoparticles or hydrogels are activated by electrical stimuli delivered from wearable or implantable devices, enabling controlled, site-specific drug release directly to tumors. This figure was generated using ChatGPT (GPT-4o-mini) and revised by the authors. The use of this AI tool is fully disclosed in accordance with journal guidelines. All rights to the generated content are retained by the authors, and the tool’s terms of use permit commercial use. (B to H) Wireless implantable electro-responsive drug delivery system integrated with wearable electronics. Reproduced with permission from Ref. [184]. (B) Schematic illustration of a severe combined immunodeficiency (SCID) in the subcutaneous region, which is wirelessly integrated with a wearable power transmitter and a wearable EEG monitoring device. Inset shows the wireless power transfer through the skin, which induces the subcutaneous drug release from the SID. (C) Image of the front side of the SID. LED, light-emitting diode. (D) Image of the backside of the SID. (E) Soft mechanical characteristics of the SID allow it to be freely twisted and bent (inset). (F) The SID can be implanted in the subcutaneous region of the mouse’s back with a minimal surgical incision. (G) Image of a wearable EEG monitoring device. (H) Image of a wearable power transmitter for the wireless power supply to the SID.

Smart electro-responsive drug carriers represent a next-generation enabling strategy that complements existing electricity-based therapies rather than functioning as stand-alone modalities. Unlike those locoregional treatments—which primarily induce direct cytotoxic effects, disrupt cellular processes, or enhance membrane permeabilization and drug transport—these systems enable electrically controlled, on-demand drug release with high spatiotemporal precision (Fig. 5A).

Against this background, smart electro-responsive drug carriers offer distinct advantages by enabling controlled and site-specific drug release in response to external electrical stimuli (Fig. 5B to H). These systems can be engineered to respond to defined electrical parameters, allowing precise modulation of drug release kinetics while minimizing systemic toxicity [183,184]. By decoupling drug release from passive diffusion and electroporation-mediated transport processes, electro-responsive carriers introduce an additional layer of control over when and where therapeutic agents are released [185].

Recent advancements have focused on the development of electro-responsive hydrogels and nanoparticles (Table S7).

For instance, polypyrrole-based nanoparticles have been synthesized to load anticancer agents like crocin [186], allicin [187], hydrogen peroxide, chloroauric acid, and ferric chloride [188], releasing the drug in response to electrical cues, which allows for targeted therapy with reduced impact on healthy tissues. Additionally, the integration of these smart carriers with wearable [1,189–193] or implantable devices [194–202] has opened new avenues for personalized cancer treatment. Such systems can be programmed to deliver drugs at specific times or in response to physiological changes, offering a dynamic approach to chemotherapy [202–204].

Moreover, hybrid systems combining electro-responsive carriers with other stimuli-responsive mechanisms have shown promise [205]. For example, hydrogels that respond to both electric fields and pH changes have been developed to exploit the acidic tumor microenvironment, ensuring that drug release occurs preferentially at the tumor site [206–208]. Such multiresponsive systems enhance the specificity and efficiency of drug delivery, reducing off-target effects [209].

Despite the promising potential, challenges remain in the clinical translation of electro-responsive drug carriers. Issues such as biocompatibility, scalability of manufacturing, and precise control over drug release kinetics are areas of ongoing research [210]. Nevertheless, the continued interdisciplinary efforts in materials science, bioengineering, and oncology are expected to overcome these hurdles, paving the way for more effective and patient-friendly cancer therapies [211,212].

Most smart electro-responsive drug carriers are still in the experimental or preclinical research stage, with limited clinical translation so far. The majority of studies—including those on electro-responsive hydrogels and micelle-based systems—have demonstrated promising results in vitro and in vivo, but not yet in routine human clinical practice.

### Future perspectives and emerging strategies in electricity-based cancer therapy

Looking forward, the future of electricity-based locoregional cancer therapies is increasingly shaped by innovation in materials science, device engineering, and systems biology. Development of wearable and implantable electro-stimulating devices [213], electro-responsive nanocarriers [214–217], and AI-driven treatment personalization platforms promises to enhance precision, minimize invasiveness, and improve patient outcomes [192,218].

Combining these therapies with real-time biosensing, controlled-release systems, and next-generation immunotherapies may lead to closed-loop cancer treatment models [219]. Additionally, integration with 3-dimensional tumor modeling and electrophysiological mapping could refine treatment planning and optimize outcomes [220]. As interdisciplinary research accelerates, electricity-based locoregional therapies are poised to become integral components of next-generation cancer care, as summarized in Table 1.

**Table 1.** Emerging technologies and prospects in electricity-based locoregional cancer therapies

Technology	Description	Key prospects	References
Combination therapies	Bioelectric modulation combined with chemotherapy, immunotherapy, or hyperthermia to enhance efficacy and target tumor bioelectricity.	Synergistic effects; increased drug uptake; improved survival; reduced toxicity; multifaceted tumor targeting.	[11,19,26,223–225]
AI-guided electroporation systems	Integration of AI with electroporation to automate and optimize delivery of therapeutic agents at the single-cell level.	Automated, reproducible, high-throughput delivery; real-time analysis; improved precision and efficacy.	[19,192,226–228]
Wireless electrotherapy devices	Minimally invasive, wearable or implantable devices delivering electromagnetic fields, light, or electric currents to tumors.	At-home or self-powered treatment; dual mechanisms (e.g., PDT + immunotherapy); targeted delivery; patient-centric.	[180,213,229–234]
Bioelectronic interfaces	Devices enabling bidirectional communication between tissues and electronics for precise, nonthermal cancer therapy.	Real-time, closed-loop control; highly localized treatment; reduced off-target effects; personalized interventions.	[235–240]
Electric field-activated nanomedicine	Nanoparticles or nanoantennas are activated by electric fields to control drug release and disrupt tumor microenvironments.	Enhanced drug delivery; selective apoptosis; minimal toxicity; amplification of TTFields; overcoming resistance.	[234,241–246]

## Conclusion

Electricity-based locoregional cancer therapies and their associated drug delivery technologies are reshaping oncology by enabling precise tumor targeting and immune modulation. Techniques such as IRE, TTFields, RFA, and smart electro-responsive carriers allow for site-specific drug delivery and controlled release, minimizing systemic toxicity while enhancing therapeutic efficacy. These approaches also promote ICD, stimulating systemic antitumor immune responses and synergizing with immunotherapies such as checkpoint inhibitors and cancer vaccines, especially in immunologically “cold” tumors.

Clinical trials in recent years have reinforced the translational promise of these approaches. Studies investigating IRE, ECT, and electro-immunotherapy have shown encouraging outcomes in various cancer types. Meanwhile, emerging innovations such as wearable electro-stimulating devices, multimodal nanocarriers, and patient-specific electro-immunotherapeutic combinations hint at a future of more personalized, adaptable cancer care.

Despite this progress, challenges remain in optimizing biocompatibility, standardizing protocols, minimizing off-target effects, and identifying robust biomarkers to guide patient selection. Continued interdisciplinary collaboration among oncologists, bioengineers, immunologists, and materials scientists is essential to fully unlock the clinical potential of these technologies. As research advances, electricity-based locoregional cancer therapies and drug delivery systems are poised to become indispensable tools for achieving more effective, personalized, and immune-integrated cancer care.

## Data Availability

Data will be made available on request.

## Supplementary Materials

Supplementary 1 Tables S1 to S7 References [247–283]
