# Supplementary material for: Electricity-Based Locoregional Cancer Therapies and Their Associated Drug Delivery Technologies: From Principles of Action to Clinical Implications
Source: Biomater Res. 2026 Jul 7;30:0386. doi: 10.34133/bmr.0386 (PMC13483372; doi:10.34133/bmr.0386)
Supplement: Supplementary file 1 [file bmr.0386.f1.doc]

***Supplemental Materials***

Title

Electricity-Based Locoregional Cancer Therapies and Their Associated Drug Delivery Technologies: from Principal of Action to Clinical Implications

**Authors**

Ngoc-Thuan Truong^1^, Arjaree Jobdeedamrong^1^, Hye-Jin Yoo^1^, Ratchapol Jenjob^1^, Seokyoung Bang^2,*^, and Su-Geun Yang^1,*^

**Affiliations**

^1^Department of Biomedical Science, BK21 FOUR Program in Biomedical Science and Engineering, Inha University College of Medicine, Incheon 22212, Republic of Korea.

^2^Department of Biomedical Engineering, Dongguk University, Goyang 10326, Republic of Korea

^*^Address correspondence to:

Seokyoung Bang; [seokyoung.bang@dongguk.edu](mailto:seokyoung.bang@dongguk.edu)

Su-Geun Yang; [sugeun.yang@inha.ac.kr](mailto:sugeun.yang@inha.ac.kr)

**Graphical abstract**

Table S1. An overview of IRE in clinical trials

| Cancer type | Method | Study design/Phase | Outcome | Reference/ *clinicaltrials.gov* |
| --- | --- | --- | --- | --- |
| Lung metastases | IRE + Radiation | Pilot, early phase 1 | Trial is ongoing to evaluate safety and feasibility of combining electrical fields with radiation. | NCT05555342 |
| Pancreatic cancer (LAPC) | IRE + Pembrolizumab | Phase 1 | Aims to evaluate safety, feasibility, and immune response potentiation of combined treatment. | NCT06378047 |
| Pancreatic cancer (LAPC) | IRE + Anti-PD-L1 | Observational | Intended to assess real-world outcomes of combined IRE and immunotherapy; results pending. | NCT06677762 |
| Pancreatic cancer (unresectable) | IRE (NanoKnife) | Single-arm observational | Observational outcomes study to evaluate safety and tumor control post-IRE. | NCT06937996 |
| Colorectal liver metastases | IRE vs. SBRT | Phase 2/3, randomized | Evaluating local control rate of IRE vs SBRT in challenging anatomical areas. | NCT06185556 |
| Metastatic colorectal cancer | IRE + CAEP + Pembrolizumab | Phase 2, prospective | Designed to assess immune sensitization by IRE and CAEP before checkpoint blockade. | NCT05609656 |
| Prostate cancer (intermediate-risk) | IRE + SBRT | Feasibility and safety | Feasibility trial evaluating synergistic effects and safety of SBRT followed by focal IRE. | NCT05345444 |
| Prostate cancer (older patients) | IRE | Observational | Showed that IRE is safe and effective for elderly patients with low-to-intermediate risk prostate cancer. | [72] |

*SBRT: stereotactic body radiotherapy CAEP: calcium electroporation*

**Table S2. An overview of TTFields in clinical trials**

| Cancer type | Method | Study design/Phase | Outcome | Reference *clinicaltrials.gov* |
| --- | --- | --- | --- | --- |
| Glioblastoma (recurrent) | TTFields vs. chemotherapy | Phase 3 | Median OS: 6.6 vs. 6.0 months | NCT00379470 [102] |
| Glioblastoma (newly diagnosed) | TTFields + temozolomide vs. temozolomide alone | Phase 3 | Median OS: 20.5 vs. 15.6 months | NCT00916409 [103] |
| Mesothelioma (unresectable) | TTFields + pemetrexed + cisplatin/carboplatin | Phase 2 | Median OS: 18.2 months | NCT02397928 [104] |
| Ovarian cancer (recurrent) | TTFields + paclitaxel | Phase 2 | Median PFS: 8.9 months | NCT02244502 [105] |
| Pancreatic cancer (locally advanced) | TTFields + gemcitabine ± nab-paclitaxel | Phase 2 | Median OS: 14.9 months | NCT01971281 [106] |
| Non-small cell lung cancer (brain metastases) | TTFields + supportive care vs. supportive care alone | Phase 3 | Median time to intracranial progression: 21.9 vs. 11.3 months | NCT02831959 [107] |
| Glioblastoma (real-world data) | TTFields + standard of care | Observational | Median OS: 19.6 months; 4-year OS: 27.7% | NCT03258021 [108] |

*OS: overall survival*

**Table S3.** **An overview of RFA in clinical trials**

| Cancer type | Method | Study design/Phase | Outcome | Reference/ *clinicaltrials.gov* |
| --- | --- | --- | --- | --- |
| Pancreatic cancer | RFA ± anti-PD-L1 immunotherapy | Preclinical (mouse models) | RFA reduced tumor growth and enhanced anti-PD-L1 efficacy; induced stromal remodeling and abscopal effects | [127] |
| Breast cancer (≤1.5 cm) | RFA | Phase 2 (RAFAELO study) | 98.6% 5-yr IBTR-free survival; minimal adverse events | [128] |
| Papillary thyroid microcarcinoma | Ultrasound-guided RFA | Prospective multicenter | High ablation success (95–98%); minimal recurrence; improved QoL vs. surgery | [129] |
| Papillary thyroid microcarcinoma (PMTC) | RFA | Prospective clinical trial | 95.9% complete tumor disappearance; low complication rates; supports RFA as a viable alternative to surgery. | NCT03432299 [130] |
| Hepatocellular carcinoma | RFA | Phase N/A | Evaluating local tumor progression rate at 12 months post-RFA; study ongoing. | NCT05449873 |
| Hepatocellular carcinoma | RFA | Phase N/A | Investigating therapeutic outcomes of anatomical RFA for peripherally located small HCC; study ongoing. | NCT05580224 |
| Hepatocellular carcinoma | RFA + Radiotherapy | Phase 3 | Studying short-term and long-term effects of combined therapy in HCC treatment; study ongoing. | NCT04202523 |
| Bile duct cancer | RFA vs. PDT | Phase 4 | Comparing survival outcomes between RFA and PDT in non-resectable bile duct cancer; study ongoing. | NCT05551299 |

**Table S4.** **An overview of ECT in clinical trials**

| Cancer type | Method | Study design/Phase | | Outcome | | Reference | |
| --- | --- | --- | --- | --- | --- | --- | --- |
| BCC, cSCC, melanoma (skin) | ECT (bleomycin, electroporation) | | Single-arm phase 2, prospective | 51% complete response, 49% partial response at 1 month; BCC had highest CR; main AE: pain (65%) | [152] | |  |
| Multiple nonmelanoma skin cancers | ECT (bleomycin) | | Prospective, comparative | ECT effective and well-tolerated; improved QoL; low toxicity | [171] | |  |
| Cutaneous/subcutaneous melanoma metastases | ECT (bleomycin, i.v) | | Single-center, retrospective | 48.3% CR, 38.2% PR, 67.5% objective response; well tolerated, main AE: mild pain | [172] | |  |
| Head and neck cancer (primary/metastatic) | ECT (bleomycin, cisplatin) | | Systematic review (multiple trials) | OR rates 82–84%, CR rates 47–59%; safe, effective, even in advanced/refractory cases | [173] | |  |
| Perihilar cholangiocarcinoma (PHCCA) | Percutaneous ECT (bleomycin) | | Pilot, prospective | 2 CR, 1 “restoration ad integrum” of liver, high local efficacy, no hepatic recurrence | [174] | |  |
| Pancreatic cancer (locally advanced) | ECT + systemic chemotherapy | | Multicenter, registry | Median OS 30.7 months, PFS 22.8 months, TTP 27.3 months; safe, encouraging survival | [175] | |  |
| Metastatic skin lesions (calcium ECT vs. standard ECT) | Calcium electroporation vs. ECT (bleomycin) | | Phase 2, randomized | OR: calcium ECT 72%, standard ECT 84%; similar efficacy, less hyperpigmentation with calcium ECT | [176] | |  |

*QoL: quality of life	CR: complete response	PR: partial response	TPP: time to response AE: adverse event	PFS: progression-free survival*

**Table S5. An overview of iontophoresis in preclinical**

| Cancer type | Method | Study design/Phase | Outcome | Reference |
| --- | --- | --- | --- | --- |
| Pancreatic cancer | Implantable iontophoretic device + Gemcitabine, Cisplatin | Preclinical (mouse models) | Significant tumor regression, reduced systemic exposure, improved tumor control and survival | [15] |
| Pancreatic cancer | Implantable iontophoretic device + FOLFIRINOX | Preclinical (mouse models) | ~10x higher tumor drug concentration vs. IV, lower plasma levels, greater tumor regression, better tolerability | [16] |
| Skin cancer | Transdermal iontophoresis + Various chemotherapeutics | Ex vivo (human skin) | Enhanced local delivery, increased tumor cell death | [143] |
| Retinoblastoma | Ocular iontophoresis + Various chemotherapeutics | Preclinical (mouse models) | Improved local drug delivery to ocular tumors | [143] |
| Breast cancer | Microfluidic iontophoresis-on-chip + Carboplatin | Preclinical (microfluidic on-chip model) | Increased drug delivery and tumor cell death in in vitro models | [188] |

**Table S6. Comparison of established electricity-based locoregional cancer therapies and emerging electro-responsive drug delivery strategies.**

| Modality | Functional role | Primary mechanism | Key advantages | Limitations | Translational challenges |
| --- | --- | --- | --- | --- | --- |
| IRE | Tumor ablation / membrane permeabilization [77] | Irreversible electroporation leading to non-thermal cell death [77] | Preserves vessels and ducts; suitable for complex anatomical sites [77] | Heterogeneous electric field distribution [138]; incomplete ablation [200] | Device optimization; treatment planning [201] |
| RFA | Tumor ablation (thermal) [21] | Joule heating via alternating current [21] | Clinically established; effective tumor debulking [116] | Heat-sink effect [118, 139]; collateral damage [202] | Limited precision near vessels [32, 116] |
| TTFields | Tumor growth inhibition [203, 204] | Alternating electric fields disrupt mitosis [25, 203] | Non-invasive; systemic applicability [205] | Prolonged treatment duration [109] | Patient compliance; treatment cost [206] |
| ECT | Drug uptake enhancement and cytotoxic therapy [23, 207] | Reversible electroporation facilitating intracellular drug delivery [23] | Enhances chemotherapeutic efficacy [208]; localized treatment [142] | Limited penetration depth [142, 209]; drug-dependent response [210] | Protocol standardization [207] |
| Iontophoresis | Drug transport enhancement[190, 211] | Electric field-driven movement of charged molecules across tissues [190, 211] | Non-invasive; localized delivery [143] | Limited penetration in dense tumors [211] | Tissue variability; parameter optimization [185] |
| Smart electro-responsive drug carriers (emerging strategies) | Electrically triggered drug release [212, 213] | Stimuli-responsive materials enabling on-demand drug release [28] | Precise spatiotemporal control; reduced systemic toxicity [212] | Limited clinical validation [214] | Scalability; biocompatibility [215] |

**Table S7. An overview of smart electro-responsive drug carriers in preclinical**

| Cancer type | Method | Study design/Phase | Outcome | Reference |
| --- | --- | --- | --- | --- |
|  |  |  |  |  |
| Hepatocellular carcinoma | Thermo-/pH-responsive injectable hydrogel | In vivo (mouse) | Sustained DOX release, significant tumor suppression | [222] |
| Esophageal cancer | pH-responsive self-assembled peptide hydrogel | In vitro/ in vivo (mouse) | Sustained, pH-triggered DOX release; enhanced tumor targeting, reduced systemic toxicity | [223] |
| Seizures (neuro) | Soft implantable drug delivery device, wirelessly controlled by wearable | In vivo (mouse)/ ex vivo | On-demand, localized drug delivery; precise seizure control; wireless integration with wearable | [220] |
| Breast cancer | Wearable ultrasound microneedle patch | In vivo (mouse) | Boosts anti-tumor immunity and anti-PD-1 synergy with minimal toxicity | [216] |
| Breast cancer | Implantable self-powered therapeutic pellet (wireless PDT/SDT hybrid therapy) | In vivo (mouse) | 85% tumor inhibition rate; recurrence suppression via ultrasound-powered LED (PDT) and SDT synergy | [224] |
| Hepatocellular carcinoma | pH-responsive self-healing injectable hydrogel | In vitro/ in vivo (rat) | Sustained, pH-triggered DOX release; improved tumor targeting and biocompatibility | [225] |
|  |  |  |  |  |

*PDT: Photodynamic therapy	SDT: Sonodynamic therapy*
